# Health services in Trinidad: throughput, throughput challenges, and the impact of a throughput intervention on overcrowding in a public health institution

**DOI:** 10.1186/s12913-018-2931-2

**Published:** 2018-02-20

**Authors:** Mandreker Bahall

**Affiliations:** 1 School of Medicine and Arthur Lok Jack Graduate School of Business, University of the West Indies, St. Augustine, Trinidad West Indies Trinidad and Tobago; 2 Department of Medicine, San Fernando General Hospital, San Fernando, Trinidad and Tobago

**Keywords:** Support services, Throughput, Throughput processes, Overcrowding, Hospital challenges

## Abstract

**Background:**

Throughput might be partially responsible for sub-optimum organisational and medical outcomes. The present study examined throughput and the challenges to ensuring optimum throughput in hospitals, and determined the effectiveness of a throughput intervention in reducing overcrowding in a public healthcare institution in Trinidad and Tobago.

**Methods:**

First, a literature review of throughput and its processes in relation to improving hospital care was conducted. Second, the challenges to throughput in healthcare were reviewed. Data were also collected from print media, hospital records, and the central statistical office in Trinidad and Tobago to discuss throughput and describe the throughput status in hospitals. Finally, the effect of a throughput intervention on overcrowding was determined. The intervention was implemented over six months, from October 2010 to March 2011, and comprised three stages of a five-stage throughput process: transferring patients to a specific medical ward, bedside electrocardiograms (ECG), and promptly obtaining patient investigative reports and patient files.

**Results:**

Problems with the throughput process led to prolonged delays or failures in obtaining lab reports, radiology services, ECGs, and pharmaceutical supplies, as well as inadequate social work services and other specialised services. During the throughput intervention, there was a reduction in overcrowding/overflow to 5–10 patients per day with a daily admission rate of 58. However, at post-intervention, there was increased overcrowding/overflow to 20–30 per day but fewer admissions (52 per day) i.e. similar to pre-intervention period. Additionally, there was an increase in bed complement in the department of medicine from 209 (2011) to 227 (2012). Overcrowding continued into 2016 and beyond: medical admissions in 2016 were 46.4 per day and the medical bed capacity was 327 (indicating a 44% increase in capacity from 2012).

**Conclusion:**

Hospital throughput processes are currently suboptimum. Improving specific throughput processes or targeting the greatest primary constraints might help decrease overcrowding.

## Background

Acceptable hospital performance is a much desired outcome by payers, patients, and providers. Trinidad and Tobago, though a small country, has high expectations for hospital performance because of its proximity to the United States of America (USA), the presence of trained professionals from the United Kingdom (UK) and USA, and a multitude of reports highlighting the poor performance of the health sector in terms of waiting times, overcrowding, and a lack of timely investigations and treatment. Such outcomes, however, are largely dependent on hospital efficiency, public policy [1, 2], and resource utilisation [1]. According to some researchers, it ‘would be difficult if not impossible’ to improve outputs without increasing inputs [3, 4]. Others believe that better outcomes can be achieved by using the ‘convergence model’, which is defined as ‘the integration of historically distinct disciplines and technologies into a unified whole that creates fundamentally new opportunities for life science and medical practice’ [5]. Another instrument for improving outcomes is the use of throughput interventions, which are recognised as a ‘critical success strategy’ [6] and a major indicator of outcome quality [7]. Research on throughput in the context of accidents and emergencies in many hospitals has revealed that overcrowding in the emergency department is attributed to the inability to transfer patients to wards because of poor-quality inpatient care. However, throughput analyses of inpatients are rare. In Trinidad and Tobago, throughput studies have not been done in emergency settings or among inpatients. This study attempts to fill this gap by reviewing throughput and throughput challenges for inpatients in a tertiary health care institution in Trinidad; as well as determining the effectiveness of a throughput intervention for inpatient care in reducing overcrowding.

### Throughput

Throughput, according to Little’s law, is defined as the rate at which a business can produce a product or service in a given unit of time [8]. In the context of healthcare, it can refer to the number of patients served in a unit of time [9]. It can also refer to a product (e.g. number of surgeries or eye tests conducted) [10] or an organisational process (i.e. ‘the cycling of patients through a hospital’s physical resource base’) [6]. Others define it as the sum total of actions (support services and operating systems) that are required to move a patient from admission to discharge [6]. Common throughput interventions target improvements in bed flow and lab report availability, more prompt ward electrocardiograms (ECG), and better clerical communication. While throughput is a major contributor to improving organisational outcomes, healthcare providers have largely ignored the optimisation of throughput processes [11]. The use of throughput processes combined with convergence technology such as electronic devices (iPods, smart phones) can further improve organisational outcomes and throughput times or length of stay (LOS) [12], which is defined ‘as the time from patient arrival to discharge time’ [12]. Well-designed throughput processes improve clinical outcomes and patient satisfaction, and decrease overcrowding. Indeed, merely increasing inputs by expanding capacity, staff, and bed occupancy [13] might not lead to the desired outcomes. Throughput partially determines high-quality healthcare [14], and encompasses the processes involved in patient flow from preadmission through discharge [6]. Throughput services (e.g. lab, radiology, pharmacy, cardiology, gastroenterology, neurology, attendant and clerical services) and prompt dispatch to the ward [15], which improves the efficiency of the emergency department, are also relevant for admitted patients. According to Press Ganey, negative organizational outcomes demonstrated by long waiting times, overcrowding, misplacement of patients, delayed surgeries, backlog, and cancellation of cases result from inadequate support or throughput services [16]. A study conducted by Pedroja found that poorly managed patient flow (i.e. overcrowding in emergency departments, intensive care units, or other hospital departments) results largely from ‘support services such as laboratory and radiology being unable to keep up, resulting in physicians having less time to focus on individual patients’ [17].

**Fig. 1:**
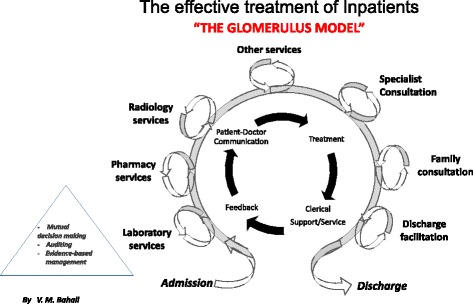
Support and Operating Systems for Effective Inpatient Treatment. Taken from: Bahall M. Health reform and the problem of hospital overcrowding: An empirical Caribbean case analysis. Soc Sci J Uni West Indies. 2014;63(2)

Throughput optimisation has its genesis in the theory of constraints (TOC), which is based on the ideology that a chain is only as strong as its weakest link. The TOC is about ‘managing the flow of a good or a person through a system and not about managing the capacity within a system’. TOC views every organization as ‘a chain of interdependent events (or processes) where the performance of each event (or process) is dependent upon the previous event’ [18]. However, maximising the efficiency of a microsystem at the expense of the macrosystem decreases organisational outcomes [11] and should be avoided. Instead, the focus should be on improving the efficiency of primary constraints, and not non-constraints. A focus on the latter could lead to ‘efficiencies syndrome’, whereby the efficiency of non-constraints is increased, thus reducing the efficiency of the entire system [18]. Management of primary constraints is key to the attainment of good patient outcomes [19]. Organisational or patient outcomes have been shown to improve with throughput interventions such as patient flow logistics [20], pharmacist-facilitated medication reconciliation [21], hospital co-ordinator assistance of patients from admission to discharge [22], patient communication [23], patient flow [24, 25], and bridging gaps identified through quality improvement models such as Six Sigma [26]. These are all represented in the revised model for hospital efficiency shown in Fig. 1. Emergency care depends heavily on emergency departures and partnering effectively with inpatient care providers to decrease emergency department boarding [27]. To improve hospital efficiency and patient outcomes, it may not be enough to choose any throughput intervention – rather, the ‘right throughput’ intervention must be chosen. This is similar to the deletion of wasteful processes and the adoption of lean techniques to improve emergency department outcomes [28]. ‘In a hospital environment characterized by increasing patient demand, constrained physical resources and a rising cost of capital, optimizing inpatient throughput is an essential operations management strategy’ [6].

### The case of Trinidad and Tobago

Trinidad and Tobago is a small twin-island state comprising an area of 1864 mile^2^ and a population of 1.33 million people, of which 35.4% are East Indian, 34.2% African, and 22.8% mixed [29]. Public health services are free. The last few decades have seen increases in inputs (staff, material, and financial resources [30]) into the healthcare system. However, despite increases in the gross domestic product per capita, health budget per capita, healthcare personnel per capita, and investments in socio-economic parameters such as education, roads, telephones, and the Internet, national health indicators have shown no significant improvement [30]. Additionally, the increasing health budget per capita has done little to curb major illnesses such as ischaemic heart disease (Table 1).

**Table 1:** Economic indicators and ischemic heart disease (IHD) mortality rate in Trinidad and Tobago

Year	GDP per capita (international US$)	Health exp. (% of total GDP)	IHD mortality per 100,000
1995	9224.7	5.1	208.5
2000	14,373.4	4.2	162.8
2005	23,287.7	5.3	244.6 (2004)
2010	27,387.9	5.3	n.a.
2014	32,083.8	5.9	301.07 (2015)

Data retrieved from [79]

Trinidad and Tobago’s health services have been plagued with inefficiencies over the last few decades, partly emanating from poor throughput processes. Reports from numerous commissions, hospital administrators, and healthcare providers have alluded to hospital inefficiencies and poor throughput processes. In 1970, the editorial of a daily newspaper reported that a full-scale enquiry into the concern was needed, since ‘the crisis in the nation’s health services seems to be getting dangerously close to the point of total collapse’ [31]. Such headlines have not been uncommon in Trinidad and Tobago. In 2016, the Trinidad and Tobago Guardian reported that hospitals are at a ‘crisis point’ [32]. Furthermore, an enquiry into the tragic deaths of 14 people, led by former chief justice Sir Issaac Hyatali in 1992, reported that the deaths were due to a massive systems failure [33]. In 2008, the Gladys Gaffoor Commission of Enquiry identified a multitude of inefficient services and processes (e.g. lab, pharmaceutical, procurement, and other support services) [34]. In 2011, the Ramsoomair Enquiry at the San Fernando General Hospital (SFGH) identified a ‘failure of medical staff to recognise the massive blood loss in a timely manner, and lack of prompt and efficient intervention by both medical and nursing staff’ [35].

Despite knowledge of the problems of waiting times for treatment, clinics, lab reports, and obtaining services (e.g. cardiology, radiology), no formal scientific studies on throughput have been conducted in Trinidad and Tobago The aim of this study was to determine the throughput challenges in a tertiary public health facility in Trinidad and Tobago and the effect of a throughput intervention on hospital overcrowding.

## Methods

This was a descriptive, observational study conducted at SFGH, the only tertiary public health facility in South Trinidad. It serves approximately half a million people, predominantly Indo-Trinidadian nationals, from central and South Trinidad. At this institution, the total annual admissions amount to 52,252, of which medical admissions account for 17,245. Furthermore, the number of total clinic visits are around 178,184, with new visits accounting for 21,618 [36]. In 2010, the overall bed capacity was 654, with 209 assigned to the department of medicine. The present bed capacity is 745, of which 327 are assigned to the department of medicine. SFGH has suffered from chronic overcrowding for decades, and it persists to this day: as much as twenty to thirty medical patients are found at any given time sitting in chairs or lying in trolleys in corridors and hallways in the medical wards and/or the emergency department.

Data covering a period of about two decades were collected from customer relations, social and pharmacy services, and various commissions of enquiry. The waiting times in the radiology lab and clinic were obtained from the clinic appointment records. Further data were obtained from utilisation reports from the South West Regional Health Authority (SWRHA) and the media, and from observational analysis. Information on health budgets and populations were gathered from the central statistical office and the Ministry of Health of Trinidad and Tobago.

A throughput process intervention was conducted over 6 months (September 2010 to March 2011; Table 2). The intervention process involved transferring patients to a specific ward that would in turn allocate them to a bed (patient flow: Stage 0); recording ECGs at the patient’s bedside (Stage 1); and the facilitation of lab services, files, and other clerical work by a ward clerk assistant attached to each medical team (Stage 2). Additionally, several minor infrastructural improvements such as additional medication trolleys and drug cupboards were made. Medication facilitation (i.e. collecting and transportation of medication from the pharmacy to the ward) was partially implemented because of a shortage of pharmacists and pharmacy assistants (Stage 3). Discharge facilitation (Stage 4) was not implemented because of time and resource constraints. Implementation of stages 0, 1, and 2 required the services of ECG technicians, ward clerk assistants attached to each medical team, and pharmacy assistants. Without this intervention, ECGs and blood investigations requested on a given day would only be obtained on the following day, the day after that, or even longer (especially if requested on a Friday). Based on the TOC, these were considered primary constraints affecting the treatment and recovery of patients. With the intervention in place, however, the ECG technicians performed the requested ECGs throughout the day at the patient’s bedside. The ward clerk, as part of the medical team, assisted in a multitude of tasks, including the collection and sorting of patient reports before attaching them to patients’ files. These files would then be available for doctors conducting early morning (and sometimes daytime) ward rounds. Ward clerks also kept a master sheet of the names and housing destinations of patients belonging to their teams. Ward clerk assistants also had several other tasks such as obtaining urgent reports and patients’ files from the medical records department, and transporting blood samples or prescription sheets to the pharmacy. They provided an important link between the medical team and other support services. The objectives were to ensure prompt and easy availability of ECGs, lab reports, medications, and other support services. The ultimate aim of the intervention was to decrease overcrowding.

**Table 2:** Staff assignment for efficiency project

No.	Designation	Number	Role
1.	ECG technician/clerk	3	To operate the ECG machine
2.	Ward clerk assistant	8	To collect and sort blood reports in a timely manner and insert them in file. To obtain patient records and perform other miscellaneous clerical duties
3.	Pharmacy assistant	3 out of 8	To mediate between prescription writing, transfer to pharmacy, and collection of medication. Inform doctors of any unavailable medication, so alternative prescriptions or methods can be used on the same day.
4.	Discharge facilitator	0 out of 8	Inform patient/family of discharge. Ensure that patients receive discharge medication, discharge letters, clinic appointments, and transport, if necessary.

During the intervention, data on patient overflow, admissions, average LOS (ALOS), and occupancy rate were collected daily. Further, patients’ LOS was monitored on a daily basis. Support services, particularly radiology services, were continuously monitored and daily feedback was provided to all stakeholders, including the Minister of Health. A daily logbook for all activities was used to determine which processes were accomplished and which were not. Multidisciplinary team meetings were conducted intermittently with various staff, including pharmacists, nurses, doctors, clerks, attendants, and social workers. Data from similar months (e.g. January 2011 [during the intervention] and January 2012 [after the intervention]) were analysed to determine changes in patterns relating to overflow patients and ALOS (Table 3).

**Table 3:** ALOS, overflow patient, medical admission, and bed capacity

	Patient overflow	Total medical admission	Total admission	Percentage overflow	Medical admission as a percentage of total admissions	ALOS (days)	Occupancy rate (%)	Bed complement
Jan 10	368	1808	4332	20.4	41.7	4.9	95.2	209
Jan 11	687	1609	4593	42.7	35.0	4.9	98.8	227
Jan 16	304	1517	4806	20.0	31.6	5.9	87.1	327
Nov 16	229	1459	4603	15.7	31.7	5.8	84.4	327

This study was conducted as part of an efficiency project, coordinated by the author, and driven and approved by the chief executive officer (CEO) of the SWRHA. Data are available to the public and healthcare providers, and can be obtained through the Freedom of Information Act. Simple descriptive analysis was undertaken and is represented in tables and graphs in the following section.

## Results

### Throughput status

Sub-optimum throughput processes were identified in a number of services, such as pharmacy, laboratory, radiology, physiotherapy, attendant, medical social work, bereavement, and communication services; ECG, echocardiogram, and stress testing services; and endoscopy, colonoscopy, endocrine, and neurological tests. For instance, for pharmacy services in 2008, the percentage of prescriptions that were fully dispensed ranged from 39.3% in April to 55.6% in December; that of partially dispensed prescriptions was 19.4% in March and 39.6% in July; and that of prescriptions not dispensed was 6.8% in May and 38% in April (Table 4). When compared to the percentages obtained in 2016, there were no meaningful changes [37]. There were, however, additions to various services, such as oncology, pharmacology, and parenteral feeding services.

**Table 4:** Pharmaceutical service RHA January–December 2008 and 2016 (prescriptions and items dispensed)

Prescriptions														
Month	Fully Disp.		%		Part Disp.		%		Not Disp.		%		Total	
	2008	2016	2008	2016	2008	2016	2008	2016	2008	2016	2008	2016	2008	2016
Jan	4175	6092	46.2	45.6	2803	4413	31	33	2065	2855	22.8	21.4	9043	13,360
Feb	5073	6376	39.2	48.7	3310	4029	25.6	30.8	4564	2674	35.3	20.4	12,947	13,079
Mar	5584	6593	53.6	45.5	2025	4375	19.4	30.2	2805	3515	26.9	24.3	10,414	14,483
Apr	4946	7730	39.3	53.7	2864	4114	22.7	28.6	4791	2554	38	17.7	12,601	14,398
May	5182	7659	56.4	58.5	3378	3736	36.8	28.5	626	2935	6.8	22.4	9186	13,103
Jun	4706	5835	45.4	45.5	4000	3489	38.5	27.2	1671	3511	16.1	27.4	10,377	12,835
Jul	5002	6059	53	45	3736	4105	39.6	30.5	706	3309	7.5	24.6	9444	13,473
Aug	4663	6512	50.5	30.8	3090	8379	33.5	39.6	1480	6285	16	29.7	9235	21,176
Sep	5583	6853	54.4	52	3628	3781	35.4	28.7	1045	2554	10.2	19.4	10,256	13,188
Oct	5283	7397	55.2	53.5	3517	4092	36.7	29.6	771	2344	8.1	16.9	9571	13,833
Nov	5623	8410	54.6	63.4	3255	3671	31.6	27.7	1267	1184	12.3	8.9	10,292	13,265
Dec	5471	6937	55.6	57.6	3548	3444	36	28.6	824	1671	8.4	13.9	9843	12,052

**Fig. 2:**
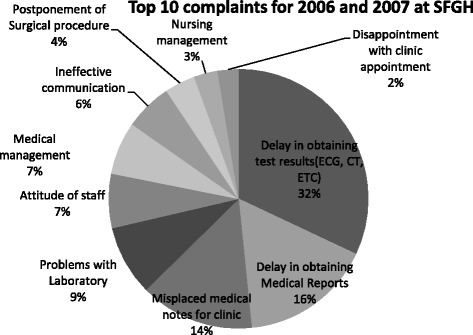
Top 10 Complaints at the Hospital (SFGH) for 2006 and 2007

There are also recurring customer complaints. At the SFGH, the most common complaints were for obtaining lab reports (32%), delays in obtaining medical reports (16%), and misplaced medical files (14%) (Fig. 2) [38]. The problem of missing files was noted by the Commission of Enquiry into the Health Sector in 2008. The enquiry revealed that ‘the records at most of the hospitals and at some clinics and health offices are very unsatisfactory. Very often, they are ‘lost or cannot be traced’ [34]. At the SFGH, delays in obtaining test results (32%) and delays in medical reports (16%) were the most frequent complaints in 2006 [38]. However, in 2015, waiting time for test results (28.9%), staff attitude (9.9%), and staff competency and credibility in relation to medical care (9.9%) were the most frequent complaints [39]. The top complaints on a national scale in 2006 were delays in obtaining test results (26%), delays in obtaining medical reports (13.3%), misplaced medical notes at clinics (11.57%), problems with the lab (7.1%), staff attitudes (5.5%), medical management (5.36%), ineffective communication (4.8%), postponement of surgical procedures (3.1%), nursing management (2.4%), and disappointment with clinic appointments (2.1%) [40]. The results also showed that processes for dealing with audits and feedback are inefficient. Complaints and feedback about monitoring and improving the healthcare delivery system do not appear to have been effectively utilised (Fig. 2). Furthermore, the 11th Annual Client Feedback Complaints and Commendations Report (2006–2007) revealed that ‘there has been little involvement of management at the Facility and Regional Health Authority (RHA) levels in resolving complaints’ [40].

Moreover, physiotherapy/radiology services continue to take days and sometimes weeks to obtain. Non-invasive cardiology services such as stress testing and echocardiography are severely compromised due to the lack of appropriate staff and resources. Many patients were not even given an appointment. Patient discharge facilitation, bereavement, and health information services were also found to be lacking. Important safety measures (e.g. isolation rooms, optimum space between beds and fire extinguishers) were either insufficient or virtually absent except for in the teaching hospital wing, which was opened in 2013.

Services that are not covered by the public healthcare system are covered by patients themselves privately, the Medical Social Work Department [34], the RHA voucher system, the Ministry of Health waiting list initiative, or not at all. Many of these services are sporadic, due to economic problems and political apathy. In fact, with the decreased budget in 2009, the RHA voucher system virtually collapsed. Needy cases were left to the Medical Social Work Department, which has very limited resources and funding, and must assist hundreds of patients with a wide range of services. Funding for the Medical Social Work Department generally comes from the Ministry of Health, although private funding is occasionally sourced. The SFGH figures for 2007–2008 reveal that limited funding was allocated and utilized for a multitude of tests. These range from about TTD 22,000 to 533,368 per month at the SFGH, and have not changed significantly over the study period. In fact, in many instances, the figures showed decreases [41]. This funding is used for a multitude of tests and services, such as bone scans, blood tests, endoscopic retrograde cholangiopancreatography, the cystic fibrosis screen test, magnetic resonance angiogram (MRA), sestamibi scans, echocardiograms, stress tests, flowmetry tests, renography, nuclear scans, magnetic resonance imaging (MRI) scans, endoscopies, and colonoscopies. Delayed or absent services lead to poor quality care and increase the cost to healthcare providers.

The Ministry of Health has developed a variety of guidelines to improve certain throughput processes, some of which are not used or will not be usable in the immediate or foreseeable future, including lab management, risk management, patient discharge guidelines, and patient transfer guidelines. ‘The Accreditation Standards Manual for the Health Sector’, prepared by the MOH of Trinidad and Tobago, recommended specific protocols, rules, and regulations to which health professionals should adhere for quality improvement. However, these systems and guidelines have also never been implemented or enforced. Other throughput processes at SFGH such as support services and waiting times, and how they compare with international benchmarks, are given in Tables 5 and 6. Importantly, dissatisfaction with support services, medication, hospitality, and management issues and customer complaints remained high [30] throughout the observation period.

**Table 5:** Operating systems and services in Trinidad and Tobago

Domain	Variable	Actual	International Benchmark
Operating and Support System and Services	Resource availability and accessibility	Inadequate and sometimes not easily accessible	Universal accessibility and availability
	Pharmacy supplies	Unable to obtain regular supply	Consistent supply made available
	Medical record system	Computerized system and database but limited usability for research	Computerized system easily accessible
	Waiting time: Services	Long delays both for inpatients (days to weeks) and outpatients (months in some cases)	Prompt; services done in a timely manner
	Lab reports	Limited investigations and delays in obtaining results (days to weeks)	Universal and timely availability
	Lab services	Inadequate and inconsistent services available	Reliable and adequate with nearly all the services available
	Bereavement room/services	Lack of rooms and services available	Sufficient rooms and services available
	Information services	Lack of reading material available	Adequate material/information readily available
	Medical social work services	Limited services and resources available to patients. Long appointment times	Adequate services/acceptable resources
	Block appointment system	Virtually non-existent at hospitals and clinics	Universal
	Protocols/quality improvement system	Virtually absent in the system and not usable	Visibly present and usable
	Audit and customer feedback	Very little feedback	Feedback ongoing on a continuous basis
	Work process	26.3% (02) strongly dissatisfied (based on employee perception survey conducted in 2002)	Generally satisfied Continuously assessed
	Investigative procedures	Lacking and unreliable	Reliable and timely
	Shuttle service/transport	Minimal use of this service	Quite good

Source: Generated by the author

**Table 6:** Customer complaints and waiting time for various services

Domain	Variable	Actual	International Benchmark
Customer complaints (percentage of total number of complaints)-from CFAR^a^	Customer complaints feedback	30.4% (delay in obtaining results), 10.4% (staff attitude), 16.3% (misplaced files), 21.5% (delay in getting appointments for ECHO and Stress tests)	Prompt response
		25.2% (wait time for outpatient clinic), 8.9% (equipment problems)	No or insignificant delay
Waiting time	Waiting time for emergency	Unpredictable (hours, sometimes > 12 h)	24% wait 4 h in emergency room (Canada)
	Waiting time for inpatient services (radiology, lab reports, and medication)	Hours, days, or weeks	Prompt, within hours.
	Waiting time for reports	Weeks, months, or years	No significant time to wait for reports
	Waiting time for operations	Months to years	Prompt to Months
	Waiting time for clinic appointments or specialist treatment	Months to years	Weeks to months

Source: Generated by the author

^a^CFAR – Client Feedback Annual Report; October 1, 2015 – September 30, 2016

### Throughput intervention results

**Fig. 3:**
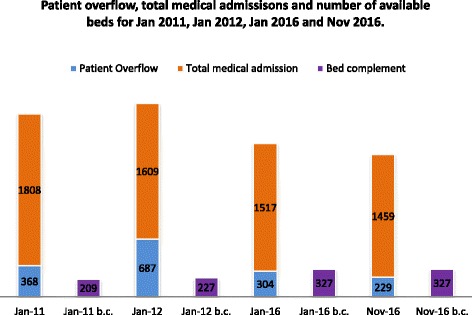
Patient overflow, total medical admissions, and number of available beds for January 2011, January 2012, January 2016, and November 2016

**Fig. 4:**
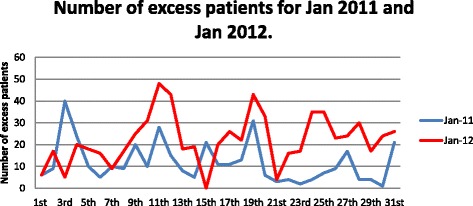
Number of excess patients for January 2011 and January 2012

From 2009 to 2010, there were 42 daily medical admissions on average, and the ALOS was 5.2. During this period, which was about 1 year before the intervention, overcrowding at the institution manifested as excess patients (between 10 and 40 patients waiting for a bed daily)? Patients were held in the corridors, waiting rooms, overflow wards, and even sent to private nursing homes. In January 2011, during the intervention, there was 368 overflow or excess patients. However, in January 2012, about 1 year after the cessation of the intervention, there were 687. This indicated an increase of 317 (or 87%) ‘excess patients’ (see Table 3 and Figs. 3 and 4). The increase in overcrowding occurred even though medical admissions decreased from 58 per day in January 2011 to 52 per day in January 2012, and while the number of medical beds increased from 209 to 227. The ALOS remained stable, at about 4.9 days, in 2011 and 2012 but increased to 5.9 in January 2016. In 2016, the number of medical beds increased to 325 (a 44% increase from 2012), with 47.6 medical admissions per day. However, overcrowding persisted, with an average of 20 overflow patients per day awaiting beds each morning.

## Discussion

Throughput at this institution is clearly suboptimum, with major gaps existing in important throughput processes, such as pharmacy, lab, and radiology services (Tables 4, 5, and 6). Throughput services, such as pharmacy [42], radiology [43], clerical [44], and lab services [45] as well as patient flow [46], are major determinants of hospital efficiency [47, 48]. As such, throughput optimisation would naturally increase efficiency [49, 50]. While increasing input is helpful, the failure to deal effectively with throughput might have contributed significantly to poor organisational outcomes such as quality of care or patient satisfaction [51], as was found in another study of SFGH patients, among whom there was a low rate of satisfaction (17.55%) with the hospital’s support services [30]. Relatively low satisfaction rates were also found for treatment (30.42%), hospitality services (27.71%), and management issues (33.99%) [30]. In 2016, the quality department found that 58% of patients were initially dissatisfied with services, and that 39% (36 out of 92) remained dissatisfied even after their complaints had been resolved [39]. Poor throughput outcome indicators such as patient safety, satisfaction, quality of hospital care, readmission rates, and human resource indicators were identified in some studies [52–54].

Given the deficient throughput services, the intervention was designed to addressing the primary constraints for patient treatment [55], including bed flow, bedside ECG, lab report availability, and access to other radiological services. The results of the intervention reveal a dramatic improvement in throughput processes, resulting in a decrease in overcrowding. In fact, when the intervention was discontinued and systems returned to the pre-intervention conditions, overcrowding worsened dramatically. In particular, the addition of beds following the intervention led to a further increase in ALOS from 4.8 to 5.8 days (25% increase) and overcrowding, which returned to pre-intervention [37] levels. This occurred despite the fewer admissions in general.

The primary constraints or throughput processes that require improvement might be unique to a particular setting and time. Therefore, it is necessary to target the ‘right throughput processes’ based on the principles of the TOC [19] and the particular healthcare setting. The primary constraints identified should then be improved within a timely manner. Ensuring appropriate throughput using principles such as the TOC has proven effective, as found in studies implementing interventions such as patient flow [56], communication [57, 58], pharmacist-facilitated discharge facilitation [21], and lab report availability [59].

The demand for better outcomes, despite limited resources and a complex environment, has been fuelled by advances in other specialities such as networking and global interconnectivity, technology, and process models. Convergence allows healthcare providers to use the contributions (e.g. operating principles) of multidisciplinary teams from various specialities (e.g. medical engineering, computer science) to improve healthcare [5]. Throughput can also benefit from the additional input brought by convergence, such as imaging, re-engineering, big data and health information technology, and nanotechnology [60]. In addition, according to the KPMJ Healthcare and Life Sciences Institute, operating processes or throughput must be adjusted to satisfy the customer base [61], and can be further facilitated with the right culture and environment [61].

Adding inputs, though helpful, is insufficient to increase outputs. It can even breed inefficiency [30, 52]. One such intervention, as demonstrated in an earlier study on increasing bed capacity to solve the overcrowding problem, only led to an increase in ALOS [62] and no significant decrease in overcrowding. Another study reported that adding more staffed beds only exacerbated the problem [63]. In fact, increasing capacity might worsen the inappropriate use of resources [13, 64]. Increasing inputs without optimising throughput (i.e. targeting the throughput processes with the highest constraints) increases costs and wastage, and decreases efficiency. Inadequate and inefficient hospital care is postulated to lead to poor services, patient dissatisfaction, and poor outcomes, which manifest as overcrowding [65, 66], increased LOS [3, 67], emergency department patient backup [68], delayed treatment [69], and heightened opportunities for errors [70]. These in turn increase costs and the risk of complications [71] and overall result in decreased hospital efficiency [72]. Furthermore, the backlog of patients in emergency departments leads to the inability to obtain appropriate beds, lost opportunities to treat, and increases in the rates of patients discharging against medical advice [73–78]. Improving hospital throughput not only improves inpatient care but frees up beds for accepting emergency patients, prevent emergency overcrowding, and improving emergency patient care.

### Limitations

This was a single-centre study. Given that the centre has its own unique culture and value systems, extrapolation to the rest of the country and other parts of the world might not be appropriate. However, similar institutions with similar backgrounds are found worldwide and the challenges and findings might be relevant to them. Another limitation is that some of the data are the author’s own observations and may be biased. In addition, the data are predominantly secondary data available to the public, and ethical approval for this study was not sought.

## Conclusion

Throughput is a critical success strategy. The throughput processes at this public health institution fall short of expectations and international benchmarks. However, the use of an appropriate throughput intervention led to a decrease in overcrowding. The underlying principles of throughput and its impact on quality outcomes can likely be applied to any public service.

## Abbreviations

ALOS: Average length of stay
DAMA: Discharging against medical advice
ECG: Electrocardiogram
ERCP: Endoscopic retrograde cholangio pancreatography
LOS: Length of stay
MOH: Ministry of Health
MRA: Magnetic resonance angiogram
MRI: Magnetic resonance imaging
RHA: Regional Health Authority
SWRHA: South West Regional Health Authority
